# The distribution and diversity of benthic macroinvertebrate fauna in Pondicherry mangroves, India

**DOI:** 10.1186/2046-9063-9-15

**Published:** 2013-08-11

**Authors:** Palanisamy Satheesh Kumar, Anisa Basheer Khan

**Affiliations:** 1 Department of Ecology and Environmental Sciences, Pondicherry University, Puducherry 605014, India; 2 Department of Biological and Environmental Sciences, University of Messina, Messina 98166, Italy

**Keywords:** Density, Diversity, Mangroves, Benthic macroinvertebrate fauna, Seasonal variation

## Abstract

**Background:**

Species distribution, abundance and diversity of mangrove benthic macroinvertebrate fauna and the relationships to environmental conditions are important parts of understanding the structure and function of mangrove ecosystems. In this study seasonal variation in the distribution of macrobenthos and related environmental parameters were explored at four mangrove stations along the Pondicherry coast of India, from September 2008 to July 2010. Multivariate statistical analyses, including cluster analysis, principal component analysis and non-multidimensional scales plot were employed to help define trophic status, water quality and benthic characteristic at the four monitoring stations.

**Results:**

Among the 528 samples collected over 168 ha of mangrove forest 76 species of benthic macroinvertebrate fauna were identified. Macrofauna were mainly composed of deposit feeders, dominated numerically by molluscs and crustaceans. Statistical analyses yielded the following descriptors of benthic macroinvertebrate fauna species distribution: densities between 140–1113 ind. m^-2^, dominance 0.17-0.50, diversity 1.80-2.83 bits ind^-1^, richness 0.47-0.74 and evenness 0.45-0.72, equitability 0.38-0.77, berger parker 0.31-0.77 and fisher alpha 2.46-5.70. Increases of species diversity and abundance were recorded during the post monsoon season at station 1 and the lowest diversity was recorded at station 2 during the monsoon season. The pollution indicator organisms *Cassidula nucleus*, *Melampus ceylonicus*, *Sphaerassiminea minuta* were found only at the two most polluted regions, i.e. stations 3 and 4. Benthic macroinvertebrate fauna abundances were inversely related to salinity at the four stations, Based on Bray-Curtis similarity through hierarchical clustering implemented in PAST, it was possible to define three distinct benthic assemblages at the stations.

**Conclusions:**

From a different multivariate statistical analysis of the different environmental parameters regarding species diversity and abundance of benthic macroinvertebrate fauna, it was found that benthic communities are highly affected by all the environmental parameters governing the distribution and diversity variation of the macrofaunal community in Pondicherry mangroves. Salinity, dissolved oxygen levels, organic matter content, sulphide concentration were the most significant parameters.

## Background

India is a large coastal nation located along the Indian Ocean with 7,517 km of coastline, along which there are many biotopes such as estuaries, lagoons, backwaters, mangroves, salt marshes, coral reefs and creeks. Mangroves in tropical and subtropical intertidal regions of the world support rich faunal resources and play an important role in estuarine and coastal food webs [1]. India has approximately 2.7% of the world’s mangroves, covering an estimated area of 4,827 sq km. Almost 80% of the mangrove forests are located along the east coast and the remaining 20% are located on the west coast [2]. The three main benthic faunal components (i.e. microfauna, meiofauna and macrofauna) represent important ecological indicators. Studies on benthic diversity, population dynamics and changes caused by natural or anthropogenic processes are essential for resource management [3]. Understanding the structure of the benthic faunal communities in relation to the impacts of pollution is an important part of monitoring changes in mangrove ecosystems in India [4-10]. Succession in macrobenthic communities, in relation to organic enrichment and pollution in the marine environment has been reported by Samidurai et al. [11]. “According to [12,13], the distribution of macrobenthic communities is highly correlated with sediment characteristics, which is related to a wider set of environmental condition”. Water quality and benthos characteristics have been investigated in coastal ecosystems around the world [10,14-16] and indicate that the health of benthic communities is related to water quality conditions in fringing communities, such as mangroves. Environmental conditions like salinity, oxygen, temperature and nutrients influence the composition, distribution and growth of biota [17]. Total organic carbon of the sediment influences fertility of the soil, thereby enhancing biological activity [18]. Diversity and density of the macrobenthos is dependent on chance settlement of pelagic larval forms of different species, affinity to suitable substratum and also the degree of stress effect caused by strong waves and tide currents. The aim of the present study was to identify the relationships between water quality and benthic macroinvertebrate fauna characteristics in Pondicherry mangroves.

## Materials and methods

### Study area

**Figure 1:**
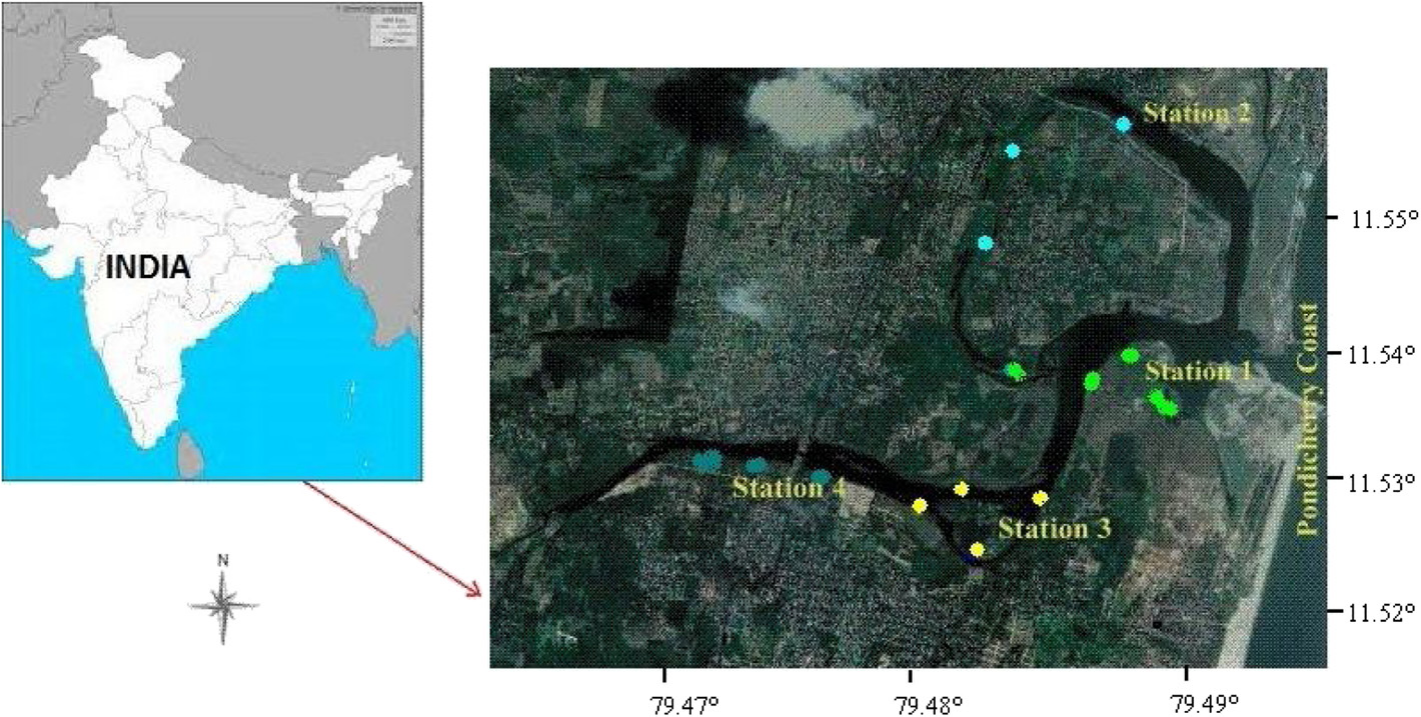
Study area of Pondicherry mangroves.

The present study area was within 11°46′03″ to 11°53′40″ North latitude and 79°49′45″ to 79°48′00″ East longitude (Figure 1). In the study region, mangroves are fringing vegetation covering 168 ha distributed along the Ariankuppam estuary, which is seasonally bar-built and has semi diurnal tides that flow eastwards and empty into the Bay of Bengal at Veerampatinam on south east coast of India, carrying the waste from the adjacent agriculture lands and industries, in addition to domestic municipal and distillery effluents. The details on GPS coordinates, zone, and soil substratum are presented in Table 1. The present investigation was carried out in four Stations: 1 Veerampattinam; 2 Thengaithittu; 3 Ariyankuppam; 4 Murungapakkam mangrove areas of the Pondicherry region. Seven true mangrove species, belonging to 3 families, and 16 other plant species, belonging to 12 families, were recorded in the study area [9,19]. Mangrove distributions include:

•*Avicennia* zone– includes a very small patch of *Avicennia marina* and densely-packed *A. officinalis* grow near the mouth region of estuary of Veerampattinam (station1),

•*Rhizophora* zone – includes four patches of *Rhizophora mucronata* and *R. apiculata* on the southern part of Thengaithittu (station 2) and four patches of *R. mucronata* and *R. apiculata* near the mouth of river.

•*Acanthus* zone – *Acanthus ebracteatus* and *A. illicifolius* forms dense stand to the western and northern side of Ariyankuppan (station 3) and Murungapakkam (station 4). *Bruguiera cylindrica* spreads from the western end of Murungapakkam up to eastern end of Ashram Islet.

•*Avicennia* and *Rhizophora* mixed zone lies near the bridge at station 4. The tides are semidiurnal and vary in amplitude from 15 to 100 cm in different regions during different seasons, reaching a maximum during monsoon and post-monsoon and a minimum during the summer. The tides are caused by a direct connection with the sea at the Veerampattinam mouth and the adjacent estuaries.

**Table 1:** Details on GPS coordinates, mangrove zone and soil substratum

**Study area**	**Mangrove zone**	**Latitude**	**Longitude**	**Substratum**
Station 1	*Avicennia* zone	11°.90′450″ N	79°.82′ 563″ E	Sand
Station 2	*Rhizophora zone*	11°.90′703″ N	79°.81′ 851″ E	Sandy and silt
Station 3	*Acanthus* + *Acicennia* mixed zone	11°.90′107″ N	79°. 80′ 547″ E	Silt and clay
Station 4	*Rhizophora* &*Acicennia* zone	11°.90′154″ N	79°.80′ 571″ E	Clay

### Sample collection and benthic macroinvertebrate fauna identification

Triplicate samples were collected every month (September 2008 to July 2010) using a metal quadrat of 25 cm × 25 cm size up to a depth of 15 cm [20]. The benthos in the sediment samples recovered after sieving through 0.5 mm mesh sieve was brought to the laboratory in polythene bags, transferred to a large, white-bottomed tray, and the animals were hand sorted. After this preliminary examination, the whole sample was treated with 5% buffered formalin and kept for further analysis. Annual rainfall, temperature and relative humidity data was obtained from meteorological department at Chennai. Dissolved oxygen was estimated by Winkler’s methods and sulphide by [21] and salinity using a refractometer. pH and temperature were measured using a pH meter, electrical conductivity (EC) was determined by using an EC (Elico) meter. Sediment texture was determined by a pipette analysis method [22]. The organic matter of the sediment was analyzed by a wet oxidation method [23]. Fauna were identified to the lowest practical taxonomic level using standard references; Polychaeta [24]; Crabs [25-27], Amphipods [28]; Mollusc [29]. For the sake of interpreting the data, a calendar year was divided into four main seasons, pre monsoon (July-September), monsoon (October-December), post monsoon (January-March), and summer (April-June). The identified samples were expressed as No/m^2^. Biodiversity indices such as species diversity, richness and evenness were calculated following standard formulae [30-33].

### Statistical analysis

All the statistical analysis methods used were according to Johnson and Wichern [34]. A variety of diversity indices have been used in benthic ecology to assess the environmental quality and the effect of disturbances on benthic communities. In the present study, calculation of two diversity indices were carried out using Shannon Wiener diversity (H’), Margalef diversity and Pielou Evenness (J’) reflects the even occurrence of species within a community. Simpson Index species richness (D) was used for univariate measures to assess community structure. Differences in univariate measures between sites were tested using correlation coefficient estimation. Correlations between the benthic macroinvertebrate fauna assemblage and physical-chemical characteristics of water and sediment were evaluated using Pearson correlation analysis. Mean and standard deviations were calculated for each parameter. These statistical analysis programs are part of the SPSS statistical program (Version 13.0 for Windows XP, SPSS, and Chicago, IL, USA). In recent years, multivariate statistical techniques are the preferred tool for a meaningful data reduction and interpretation. Multivariate statistical techniques such as cluster analysis (CA), Non-Multidimensional Scale plot (MDS) and Principle Component analysis (PCA) have widely been used as unbiased methods in analysis between water quality and marine organisms; water quality [35-37], phytoplankton characteristics [38,39], benthos characteristics [40-42]. Multivariate analysis such as, CA, MDS, and PCA were constructed based on macro faunal abundance, diversity indices and with environmental parameters. Based on the groups obtained from a cluster analysis, species that contributed the most to this distribution were determined using similarity percentage program PAST (Statistical Version 1.93 for Windows XP).

### Data treatment

Nearly all the multivariate statistical methods need variables to confirm the normal distribution, thus, the normality of the distribution of each variable was checked by analyzing kurtosis and skewness statistical tests before multivariate statistical analysis was conducted [43]. The original data demonstrated values of kurtosis ranging from −1.82 to 10.56 and skewness values ranging −1.91 to 3.61 indicating that the data was not normally distributed. Since most of values of kurtosis and skewness were >0, the raw data of all variables were transformed in the form x′=log 10(x). After transformation, the kurtosis and skewness values ranged from −1.081 to 3.01 and 1.01 to 1.66, respectively, indicating that all the data were normally distributed or close to being normally distributed. In the case of CA, PCA, and MDS, all log-transformed variables were also z-scale standardized to minimize the effects of difference units and variance of variables and to render the data dimensionless [44].

## Results

### Physical-chemical characteristics

**Figure 2:**
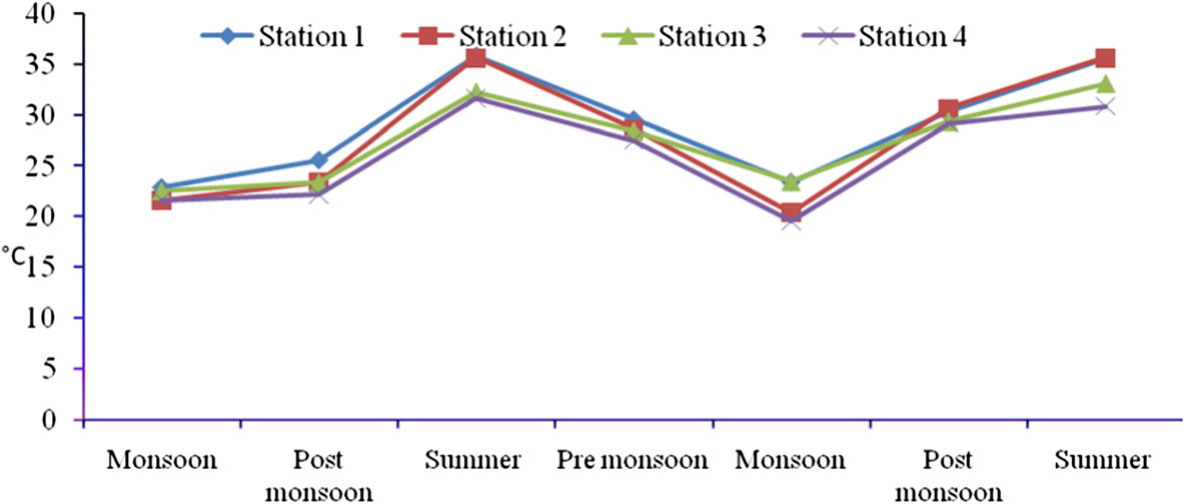
Seasonal variation of water temperature at four stations.

**Figure 3:**
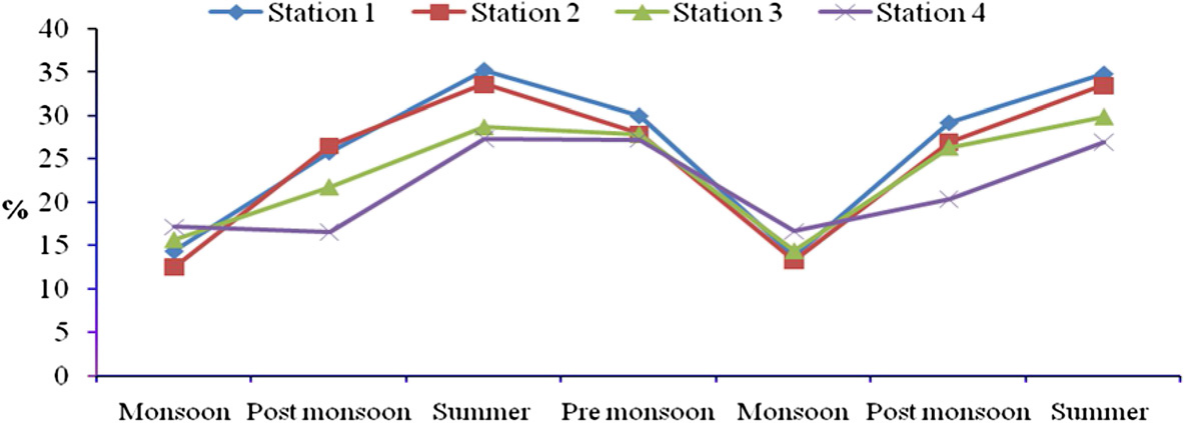
Seasonal variation of salinity at four stations.

**Figure 4:**
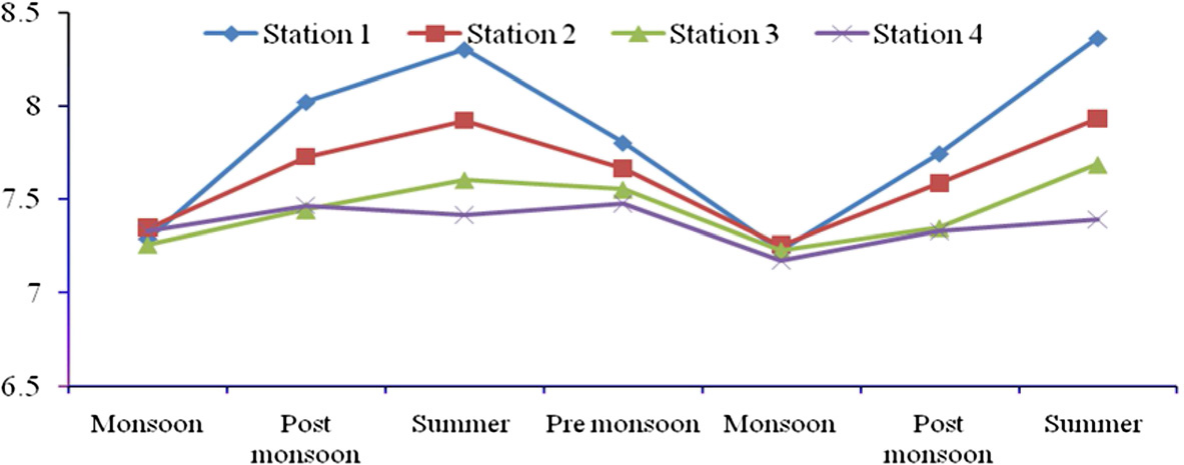
Seasonal variation of pH at four stations.

**Figure 5:**
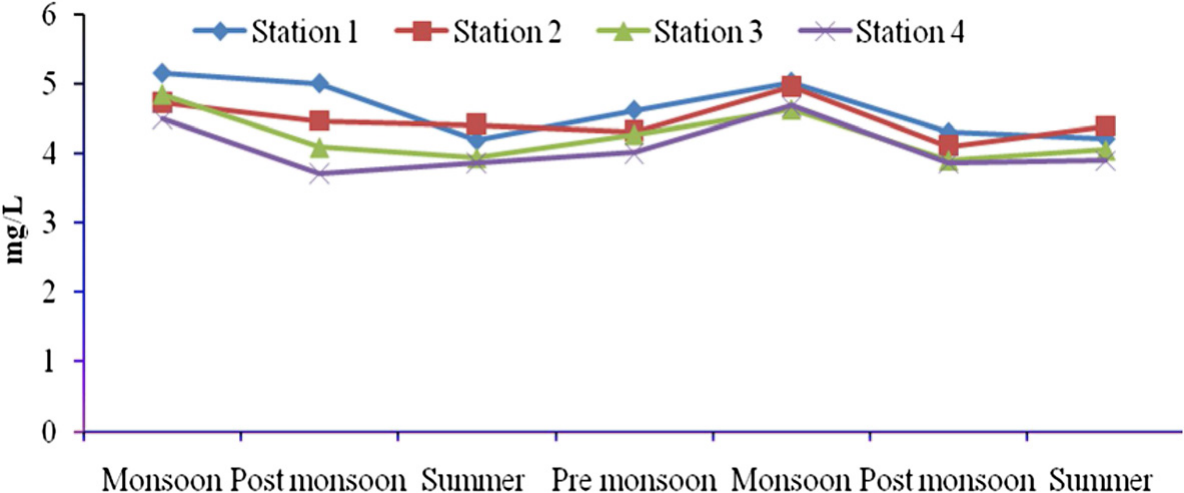
Seasonal variation of dissolved oxygen at four stations.

**Figure 6:**
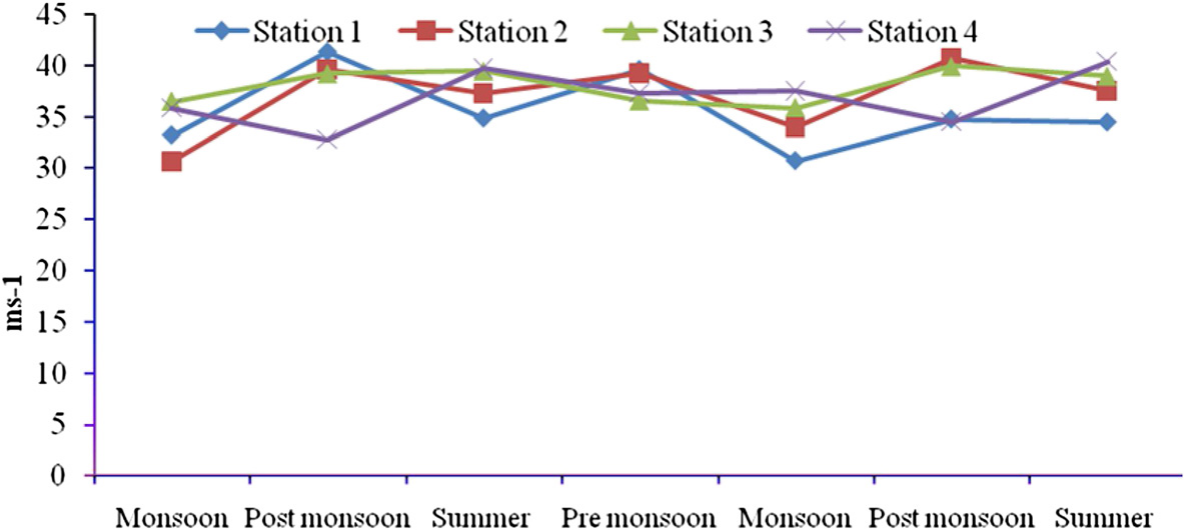
Seasonal variation of electrical conductivity at four stations.

**Figure 7:**
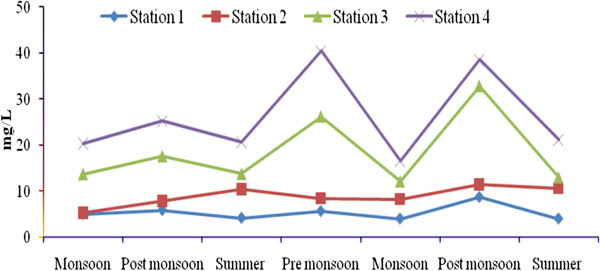
Seasonal variation of Sulphide at four stations.

Total rainfall during Sep 2008- July 2010 was 2810.9 mm, and relatively high rain fall was observed in November 2008 due to the Nisha storm (808 mm). Air temperatures ranged from 17.9-41.7°C, with minimum and maximum values in November 2008 and June 2010, respectively. The relative humidity ranged from 37–100 (%), with high values during the monsoon season (Oct-Dec) and lower values during summer season. Surface water temperatures ranged from 19.6°C-35.9°C, with the highest temperatures from April to June. A low temperature of 19.6°C was recorded during the monsoon season (Figure 2). Salinities ranged from 12.5-35.2 psu (Figure 3), and generally decreased towards the stations 2 to 4. Maximum value of surface water salinity was 35.3 psu at Station 1 during the summer and the minimum was 12.5 psu at Station 2 during monsoon season. Table 2 shows the correlation coefficient between the physical- chemical characteristics of water and sediment and benthic fauna. Significant positive correlation obtained between salinity and water temperature (r = 0.90; *p* < 0.01) indicates a seasonal component. pH ranged from were 7.11-8.36 (Figure 4). The lowest pH was 7.11 at Station 4 during the monsoon season and the highest was 8.36 at Station 1 during the summer season. Dissolved oxygen (DO) concentrations ranged from 3.71-5.16 mg/l. Stations 1 and 2 exhibited the highest DO values throughout the study period. Low DO concentrations recorded during the summer season were in part attributable to higher surface water temperature (Figure 5). Electrical conductivity at the four stations ranged from 30.60-41.33 ms^-1^. The highest EC was recorded in the post monsoon season (41.33 ms^-1^) at Station 1 (Figure 6). Sulphide concentrations ranged from 4.03- 40.43 mg/l, with the highest values during the pre-monsoon season and lowest during monsoon (Figure 7). Significant negative correlation (r = −0.617; *p *< 0.01) was observed between sulphide and DO, indicating that the DO is largely influenced by the sulphide. Ordination of environmental variables by correlation – based principal component analyses confirmed distinctions between the four sampling stations.

**Table 2:** Correlation of benthic macrobenthic invertebrate species diversity indices with environmental parameters

	**Berger**	**Clay**	**DO**	**Dominance**	**EC**	**Equitability**	**Eve**	**Fisher**	**MD**	**OM**	**pH**	**Richness**	**Salinity**	**Sand**	**H**	**Silt**	**Sulphide**	**Tem**
Berger	1.00	−0.31	0.29	0.84**	−0.28	−0.94**	−0.65**	0.68**	−0.01	−0.58**	0.27	−0.68**	0.05	0.29	−0.09	−0.10	−0.59**	0.19
Clay	−0.31	1.00	−0.46*	−0.31	0.09	0.26	0.28	−0.38*	0.12	0.15	−0.11	0.23	−0.08	−0.79**	0.11	0.17	0.22	−0.07
DO	0.29	−0.46*	1.00	0.36	−0.34	−0.26	−0.04	0.38*	−0.12	−0.49**	−0.15	−0.25	−0.48**	0.44*	−0.03	−0.15	−0.62**	−0.51*
Dominance	0.84**	−0.31	0.36	1.00	−0.26	−0.76**	−0.53**	0.65**	−0.14	−0.59**	0.07	−0.59**	−0.08	0.21	−0.15	0.07	−0.52**	0.08
EC	−0.28	0.09	−0.34	−0.26	1.00	0.34	0.35	−0.05	0.54**	0.19	0.20	0.40*	0.52**	−0.06	0.48*	−0.04	0.16	0.33
Equitability	−0.94**	0.26	−0.26	−0.76**	0.34	1.00	0.65**	−0.64**	0.15	0.61**	−0.31	0.72**	−0.05	−0.25	0.23	0.08	0.57**	−0.22
Evenness	−0.65**	0.28	−0.04	−0.53**	0.35	0.65**	1.00	−0.37*	0.28	0.11	0.05	0.89**	0.08	−0.27	0.40*	0.10	0.08	−0.15
Fisher	0.68**	−0.38*	0.38*	0.65**	−0.05	−0.64**	−0.37*	1.00	0.26	−0.64**	0.41*	−0.32	0.25	0.43*	0.22	−0.23	−0.59**	0.27
MD	−0.01	0.12	−0.12	−0.14	0.54**	0.15	0.28	0.26	1.00	−0.22	0.50**	0.44*	0.48*	−0.05	0.97**	−0.13	−0.20	0.23
Om	−0.58**	0.15	−0.49**	−0.59**	0.19	0.61**	0.11	−0.64**	−0.22	1.00	−0.38*	0.22	−0.07	−0.20	−0.29	0.10	0.73**	−0.03
pH	0.27	−0.11	−0.15	0.07	0.20	−0.31	0.05	0.41*	0.50**	−0.38*	1.00	0.20	0.82**	0.24	0.43*	−0.32	−0.39*	0.72*
Richness	−0.68**	0.23	−0.25	−0.59**	0.40	0.72**	0.89**	−0.32	0.44*	0.22	0.20	1.00	0.28	−0.26	0.53**	0.10	0.28	0.03
Salinity	0.05	−0.08	−0.48**	−0.08	0.52**	−0.05	0.08	0.25	0.48*	−0.07	0.82**	0.28	1.00	0.18	0.38*	−0.27	−0.04	0.90**
Sand	0.29	−0.79**	0.44*	0.21	−0.06	−0.25	−0.27	0.43*	−0.05	−0.20	0.24	−0.26	0.18	1.00	−0.07	−0.72**	−0.39*	0.19
H	−0.09	0.11	−0.03	−0.15	0.48*	0.23	0.40*	0.22	0.97**	−0.29	0.43*	0.53**	0.38*	−0.07	1.00	−0.08	−0.22	0.11
Silt	−0.10	0.17	−0.15	0.07	−0.04	0.08	0.10	−0.23	−0.13	0.10	−0.32	0.10	−0.27	−0.72**	−0.08	1.00	0.36	−0.26
Sulphide	−0.59**	0.22	−0.62**	−0.52**	0.16	0.57**	0.08	−0.59**	−0.20	0.73*	−0.39*	0.28	−0.04	−0.39*	−0.22	0.36	1.00	−0.03
Tem	0.19	−0.07	−0.51**	0.08	0.33	−0.22	−0.15	0.27	0.23	−0.03	0.72**	0.03	0.90**	0.19	0.11	−0.26	−0.03	1.00

*Correlated at 5% significance level.

**Correlated at 1% significance level.

*Berger* Berger Parker, *DO* Dissolved Oxygen, *EC* Electrical conductivity, *Fisher* Fisher Alpha, *MD* Margalef Diversity, *H* Shannon Weaver Diversity, *Tem* Temperature.

### Mangrove sediment characteristics

**Figure 8:**
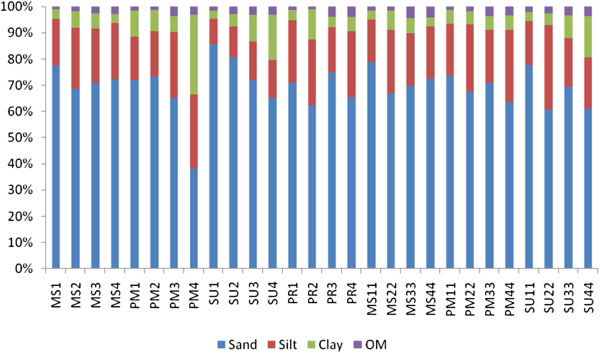
Seasonal variations of sediment characteristics at four stations.

Mangrove sediment substratum was mainly composed of sand with an admixture of silt and clay. The sand fraction ranged between (39.54-87.31%), followed by silt (9.63-32.37%), clay (3.06-31.20%) and organic matter (0.94 - 4.64%) (Figure 8). Seasonally, Station 1 recorded higher fractions of sand in the summer, higher silt content during the post-monsoon and pre-monsoon period, and higher clay during the post monsoon season at Station 4. Changes in sediment composition were mainly due to transport of sediments by tides and currents.

### Species composition

**Figure 9:**
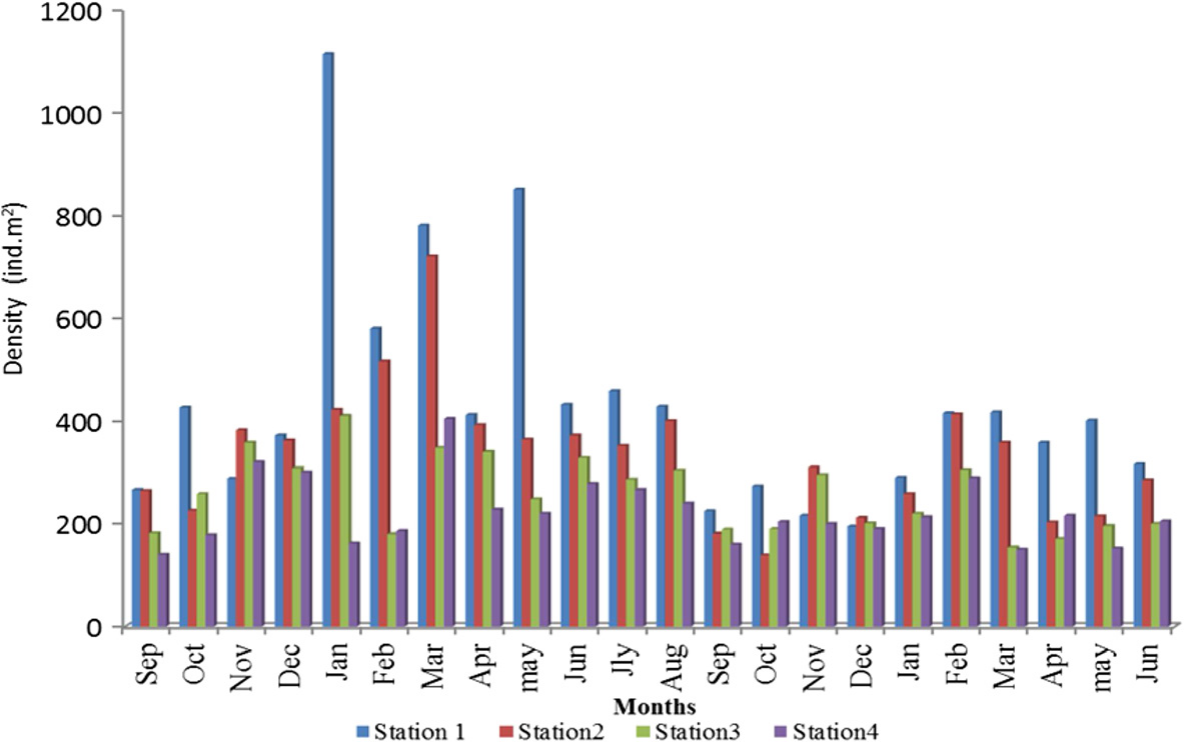
Species abundance in Pondicherry mangroves.

A total of 76 invertebrate taxa were recorded from the four mangrove stations, including 35 molluscs (16 bivalves and 21 gastropods), 22 crustaceans, 7, amphipods, 6 polychaetes, 3 barnacles and an oligochaete (Table 3). Benthic macroinvertebrate fauna densities ranged from 140–1113 ind.m^-2^ (Figure 9). By station, benthic macroinvertebrate fauna density (ind.m^2^) ranged from 193–1113, 139–720, 154–410, and 140–404 at Stations 1–4, respectively, with highest values in early post monsoon season.

**Table 3:** Checklist of benthic fauna recorded at stations 1–4, Pondicherry mangroves

**S. no**	**Species**	**Station 1**	**Station 2**	**Station 3**	**Station 4**
	**Polychaeta**				
1	*Capitella capitata*	-	-	+	+
2	*Marphysa macintoshi*	-	-	+	+
3	*Marphysa sp*	-	+	-	+
4	*Namalycastis indica*	+	-	+	-
5	*Nereis sp*	+	+	-	+
6	*Pseudonereis variegata*	-	+	-	-
	**Oligochaeta**				
7	*Pontodrillus litoralis*	-	+	-	+
	**Crustacea**				
	**Sessilia**				
8	*Balanus Amphitrite*	+	+	+	+
9	*B. variegatus*	-	+	+	-
10	*B. reticulatus*	-	-	+	-
11	*Callappa lophos*	-	+	-	-
12	*Cardisoma carnifex*	+	+	+	+
13	*Charybdis lucifera*	+	+	-	-
14	*C. feriata*	+	+	+	-
15	*C. granulta*	+	-	+	-
16	*Muradium tetragonum*	+	+	+	+
17	*Scylla serrata*	+	+	+	+
18	*S. tranquebarica*	+	+	+	+
19	*Selatium brockii*	+	+	+	+
20	*Portunus sanguinolentus*	+	-	-	-
21	*P. pelagicus*	+	+	-	-
22	*Thalamitta crenata*	+	+	-	-
23	*T. chaptali*	-	+	+	-
24	*Uca anulipes*	+	+	+	+
25	*U. triangularis*	-	+	+	-
26	*U. inversa*	-	-	+	+
27	*Metapograpsus latifrons*	+	-	-	+
28	*M. messor*	-	-	-	+
29	*Ocypode macrocera*	+	+	+	-
30	*O. platytarsis*	+	+	-	-
31	*Macropthalmus depressus*	+	-	-	-
32	*M. erato*	-	+	+	-
	**Amphipoda**				
33	*Cymadusa pathyi*	-	-	+	+
34	*Eriopisella sp*	+	-	-	-
35	*Eriopsia chilkensis*	+	-	-	-
36	*Grandidierella bonnieroides*	-	-	-	+
37	*G. pathyi*	-	-	-	+
38	*Isala montagui*	-	-	-	+
39	*Melita dentada*	-	-	+	+
	**Mollusca**				
	**Bivalvia**				
40	*Anadara granosa*	+	+	+	+
41	*A. rhombea*	+	+	+	-
42	*Bivalve spat*	-	-	+	-
43	*Crassostrea madrasensis*	+	+	+	+
44	*Cucullea cucullata*	+	-	-	-
45	*Donax faba*	+	-	-	-
46	*D. scortum*	+	-	-	-
47	*Mactra laevis*	-	+	-	-
48	*Marcia opima*	+	-	-	-
49	*Meretrix meretrix*	+	+	+	+
50	*M. casta*	+	+	+	+
51	*Perna viridis*	+	+	+	-
52	*P. indica*	+	-	-	-
53	*Scapharca inaequivalvis*	+	-	-	-
54	*Saccostrea cucullata*	+	-	+	-
55	*Modiolus metcalfei*	+	-	-	-
	**Gastropoda**				
56	*Cantharus tranquebaricus*	+	+	-	-
57	*Cassidula nucleus*	-	-	+	+
58	*Cerithidea cingulata*	+	+	+	+
59	*C. obtusa*	-	+	-	-
60	*Clithon oualaniensis*	+	+	-	-
61	*Herpetopoma sp*	+	-	-	-
62	*Melampus ceylonicus*	-	-	+	+
63	*Littorina melanostoma*	-	-	+	-
64	*Natica marochiensis*	+	+	+	+
65	*Nassarius pullus*	-	-	-	+
66	*N. stolatus*	+	+	+	-
67	*Neritina violacea*	-	-	+	+
68	*Polinices mammilla*	+	+	-	-
69	*Sinum neritoideum*	+	-	-	-
70	*Sphaerassiminea minuta*	-	-	+	+
71	*Telescopium telescopium*	+	+	+	+
72	*Thais bufo*	-	+	-	-
73	*Trochus radiatus*	+	-	-	-
74	*Turbo brunneus*	+	-	-	-
75	*Turritella attenuata*	+	-	-	-
76	*Vittina coromandeliana*	+	+	+	-

(+) Presence; (−) absence.

### Dominant taxa

Species dominance in the present study varied from 0.174 to 0.508, the minimum value of dominance was recorded in the pre-monsoon season and maximum value during the monsoon season in 2009 (Table 4). Dominance values showed significant positive correlation between the berger parker (r = 0.843; *p *< 0.01), and fisher alpha (r = 0.650; *p *< 0.01). However, there was a negative correlation between evenness (r = − 0.526; *p *< 0.01), richness (r = −0.586; *p *< 0.01) and equitability (r = −0.764; *p *< 0.01). Gastropod densities ranged from 36–333 organism m^-2^. Among gastropods, *Cerithedia cingulata* was the most dominant, followed by *Cassidula nucleus, Melampus ceylonicus, Sphaerassiminea minuta* and *Telescopium telescopium.* Bivalve densities ranged from 12–20 organism m^-2^. Among bivalves *Crassostrea madrasensis* was the most dominant, followed by *Meretrix meretrix, M. casta, Perna viridis* and *Anadara granosa.* Densities of brachyuran crabs ranged from 29–71 ind. m^2^. Among brachyuran crabs belonging to 12 genera and 5 families were recorded; crabs belonging to the families Portunidae and Ocipodidae were the most dominant, representing a total of 16 species. Six crab species are commercially important, out of which *Scylla serrata*, *Thalamitta crenata* and *Portunus sanguinolentus* are caught in large quantities from stations 1 and 2. *Portunus pelagicus*, *P. sanguinolentus* and *T. crenata* were totally absent in stations 3 and 4.

**Table 4:** Macro benthic invertebrate species diversity indices of Pondicherry mangroves

**Season**	**Dominance**	**Sha. diversity**	**Margalef diversity**	**Evenness**	**Richness**	**Equitability**	**Fisher alpha**	**Berger parker**
MS1	0.50	1.98	1.81	0.53	0.52	0.47	5.70	0.70
MS2	0.42	1.80	1.67	0.45	0.47	0.38	4.39	0.77
MS3	0.30	2.1	1.96	0.67	0.66	0.63	3.64	0.49
MS4	0.36	1.99	1.83	0.59	0.60	0.63	2.97	0.56
PM1	0.31	2.83	2.70	0.64	0.68	0.62	4.8	0.54
PM2	0.31	2.74	2.57	0.63	0.68	0.63	5.03	0.54
PM3	0.25	2.46	2.27	0.72	0.74	0.72	2.80	0.42
PM4	0.26	2.32	2.14	0.71	0.74	0.70	2.99	0.39
SU1	0.42	2.28	2.12	0.63	0.69	0.52	5.28	0.63
SU2	0.35	2.15	2.05	0.62	0.69	0.61	5.05	0.58
SU3	0.26	2.34	2.18	0.59	0.63	0.71	3.44	0.39
SU4	0.28	2.13	2.00	0.70	0.67	0.70	2.46	0.44
PR1	0.40	2.56	2.35	0.72	0.74	0.55	5.50	0.62
PR2	0.44	2.31	2.10	0.67	0.66	0.52	5.22	0.66
PR3	0.17	2.23	2.12	0.63	0.68	0.79	4.56	0.31
PR4	0.24	2.21	2.01	0.71	0.74	0.77	2.61	0.32
MS11	0.41	2.06	1.85	0.54	0.54	0.49	4.53	0.64
MS22	0.47	2.08	1.95	0.63	0.63	0.60	4.79	0.56
MS33	0.39	2.33	2.2	0.62	0.64	0.64	4.54	0.61
MS44	0.35	2.07	1.90	0.64	0.67	0.71	3.43	0.47
PM11	0.48	2.78	2.62	0.52	0.61	0.56	5.04	0.74
PM22	0.46	2.46	2.32	0.58	0.60	0.51	5.39	0.66
PM33	0.37	2.13	2.11	0.51	0.61	0.58	4.68	0.59
PM44	0.38	2.00	1.87	0.48	0.61	0.57	4.16	0.60
SU11	0.48	2.28	2.25	0.54	0.66	0.51	5.33	0.67
SU22	0.41	2.20	2.06	0.61	0.65	0.57	4.27	0.56
SU33	0.47	1.97	1.97	0.48	0.52	0.51	4.68	0.67
SU44	0.34	2.21	2.22	0.55	0.58	0.56	4.21	0.61

*MS1* Monsoon station 1, *MS2* Monsoon station 2, *MS3* Monsoon station 3, *MS4* Monsoon station 4, *PM1* Post monsoon station 1, *PM2* Post monsoon station 2, *PM3* Post monsoon station 3, *PM4* Post monsoon station 4, *SU1* Summer station 1, *SU2* Summer station 2, *SU3* Summer station 3, *SU4* Summer station 4, *PR1* Premonsoon station 1, *PR2* Premonsoon station 2, *PR3* Premonsoon station 3, *PR4* Premonsoon station 4, *MS11* Monsoon station 11, *MS22* Monsoon station 22, *MS33* Monsoon station 33, *MS44* Monsoon station 44, *PM11* Post monsoon station 11, *PM22* Post monsoon station 22, *PM33* Post monsoon station 33, *PM44* Post monsoon station 44, *SU11* Summer station 11, *SU22* Summer station 22, *SU33* Summer station 33, *SU44* Summer station 44.

**Figure 10:**
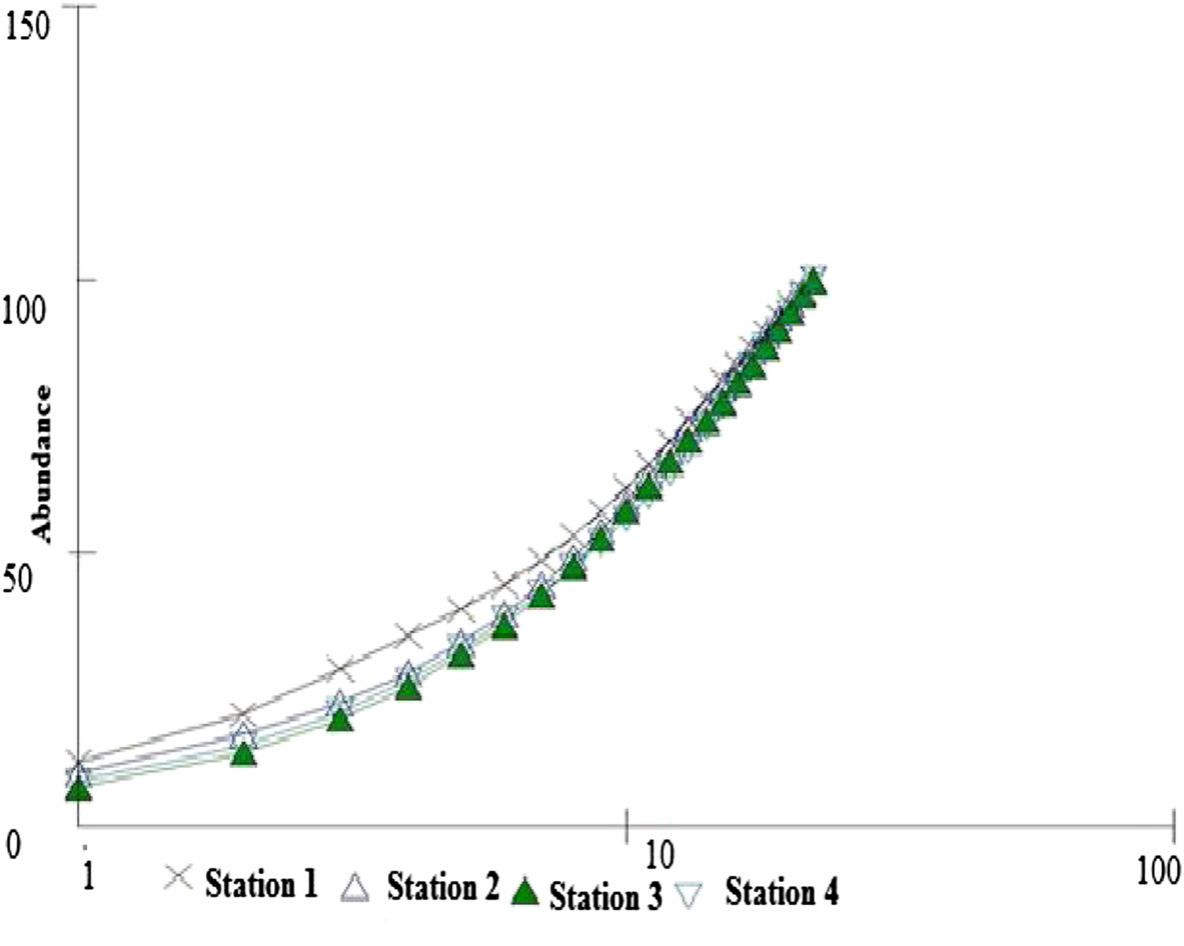
K dominance plot.

Seven species of amphipods were recorded for the first time from Pondicherry mangroves. The Gammaroidea species *Eriopisella* sp and *Eriopsia chilkensis* were collected at stations 1 and 2. The Melitidae species *M. dentada* and Ampithoidae species *Cymadusa. pathyi* were observed at stations 3 and 4. The Aoridae species *Grandidierella bonnieroides, G. pathyi* and *Isala Montagui* were observed at station 4. A *k-*dominance plot curve was drawn based on high- and low flow macroinvertebrate community data. In the present investigation the data collected during various seasons and from all four stations was fed into to the dominance plot (Figure 10). The highest dominance was seen at Station 1, where the macroinvertebrate assemblage had the highest diversity. The curve for Stations 3 and 4 showed the minimum diversity. The highest diversity was recorded in post monsoon season and lowest diversity in the monsoon season. The dominance curve did not show an ‘S’ shape due to the presence of opportunistic species *C. nucleus, M. ceylonicaus*, and *S. minuta.*

### Seasonal variation of species diversity

**Figure 11:**
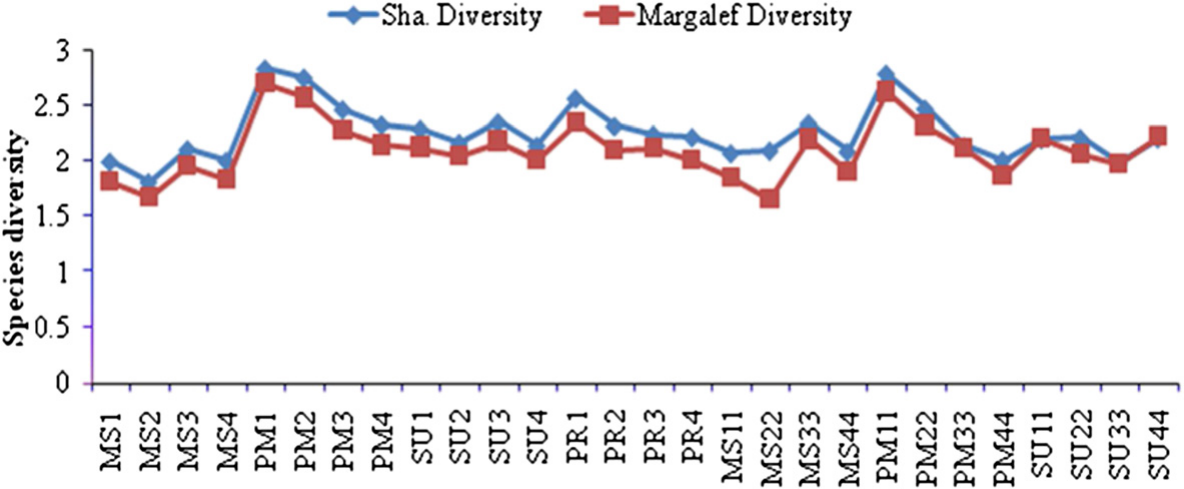
Seasonal variation of species diversity of Pondicherry mangroves.

**Figure 12:**
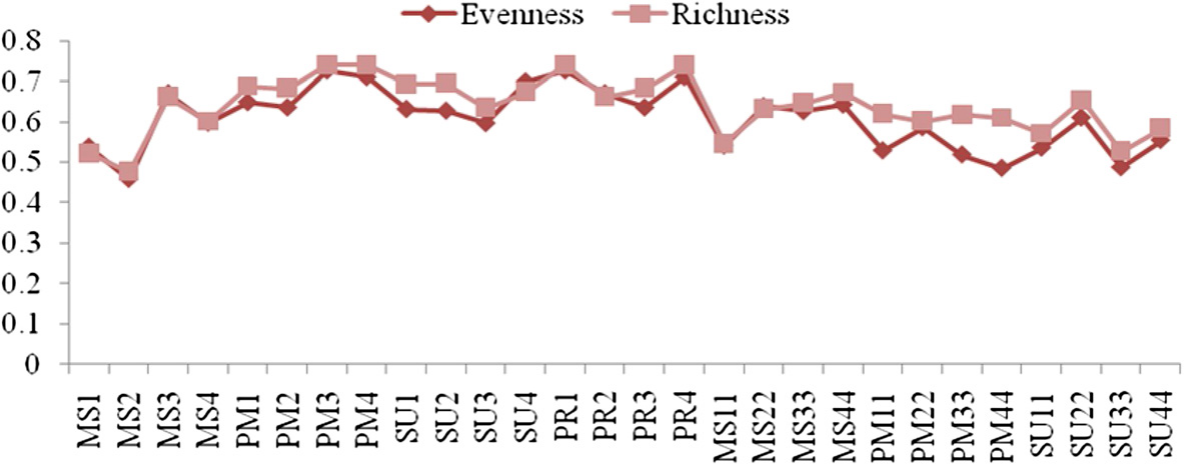
Seasonal variations of species evenness and richness.

**Figure 13:**
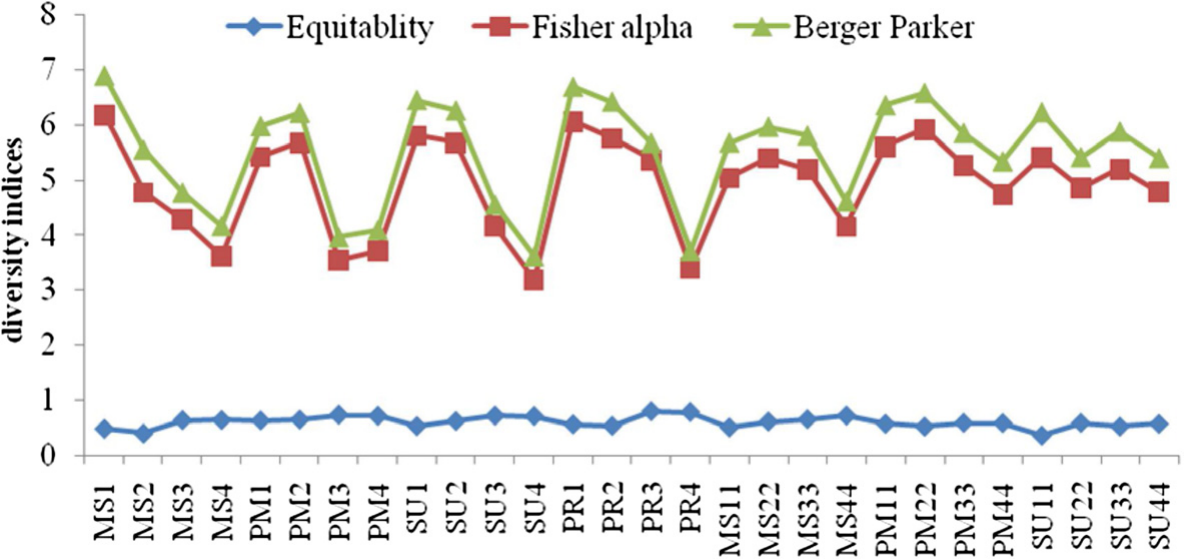
**Seasonal variations of diversity indices.** MS1= Monsoon station 1 ; MS2 = Monsoon station 2; MS3 = Monsoon station 3; MS4 = Monsoon station 4; PM1= Post monsoon station 1; PM2 = Post monsoon station 2; PM3 = Post monsoon station 3; PM4 = Post monsoon station 4; SU1 = Summer station 1; SU2 = Summer station 2; SU3 = Summer station 3; SU4 = Summer station 4; PR1 = Premonsoon station 1; PR2 = Premonsoon station 2; PR3 = Premonsoon station 3; PR4 = Premonsoon station 4; MS11 = Monsoon station 11; MS22 = Monsoon station 22; MS33 = Monsoon station 33; MS44 = Monsoon station 44; PM11 = Post monsoon station 11; PM22 = Post monsoon station 22; PM33 = Post monsoon station 33; PM44 = Post monsoon station 44; SU11 = Summer station 11; SU22 = Summer station 22; SU33 = Summer station 33; SU44 = Summer station 44.

Shannon diversity (*H’*) varied between the stations ranging from 1.80-2.83 and Margalef diversity ranged from 1.67 to 2.70. Diversity was high in the post monsoon season at Station 1 and low in the monsoon season and summer of 2009 at Station 2 (Figure 11). There was a positive correlation with Margalef diversity (r = 0.966; *p *< 0.01) and evenness (r = 0.403; *p *< 0.05). Simpson Index species richness (D) ranged from 0.47-0.74 and was highest in the post-monsoon at Station 4 and lowest in the monsoon season at Station 2 (Figure 12). Species richness was positively correlated to evenness (r = 0.89; *p *< 0.01). The Pielous’s evenness (J’) index showed spatio-temporal variation, with a minimum value during the monsoon season at Station 2 (J’=0.45), and the maximum value in pre-monsoon season at Station 1 (J’=0.72). Evenness was positively correlated to equitability (r = 0.652; *p *< 0.01). Berger Parker diversity index ranged from 0.312 – 0.772, with the highest value during the monsoon season at Station 2 and the lowest value in pre monsoon season at Station 2 (Figure 13). Equitability ranged from 0.38-0.79 and was highest during the pre-monsoon season at Station 3 and lowest in the monsoon season at Station 2. Berger Parker was negatively correlated to equitability (r = −0.944; *p *< 0.01), evenness (r = −0.653; *p *< 0.01) and richness (r = −0.653; *p *< 0.01). Fisher alpha ranged from 2.46-5.71, and was highest during the monsoon season at Station 1 and lowest in the summer at Station 4. There was a negative correlation between Fisher alpha and equitability (r = − 0.643; *p* < 0.01).

### Relationship between benthic fauna and environmental factors

Correlation analysis showed a relationship between benthic diversity indices and abiotic variables (Table 2). Among the independent variables analyzed, there was a significant positive correlation between salinity, benthic faunal diversity (r = 0.381; *p *< 0.05) and Margalef diversity (r = 0.477; *p *< 0.05). Among the physical environmental variables, pH and EC were positively correlated with Shanon (*p *< 0.05), Margalef (*p *< 0.01) diversity. DO was positively correlated with Fisher alpha (r = 0.425; *p *< 0.05). Sulphide concentration was positively correlated with equitability (r = 0.571; *p *< 0.01). Organic matter (OM) content of sediments was negatively correlated with species dominance (r = −0.586; *p *< 0.01), Fisher alpha (r = −0.645; *p *< 0.01) and Berger Parker (r = −0.584; *p *< 0.01). Sulphide concentration was negatively correlated with species dominance (r = −0.519; *p *< 0.01), Berger Parker (r = −0.586; *p *< 0.01), and Fisher alpha (r = −0.588; *p *< 0.01). Sand content of sediments was positively correlated with the Fisher alpha (r = 0.425; *p *< 0.05). Clay content was negatively correlated with Fisher alpha (r = −0.384; *p *< 0.05).

### Multivariate statistical analysis

**Figure 14:**
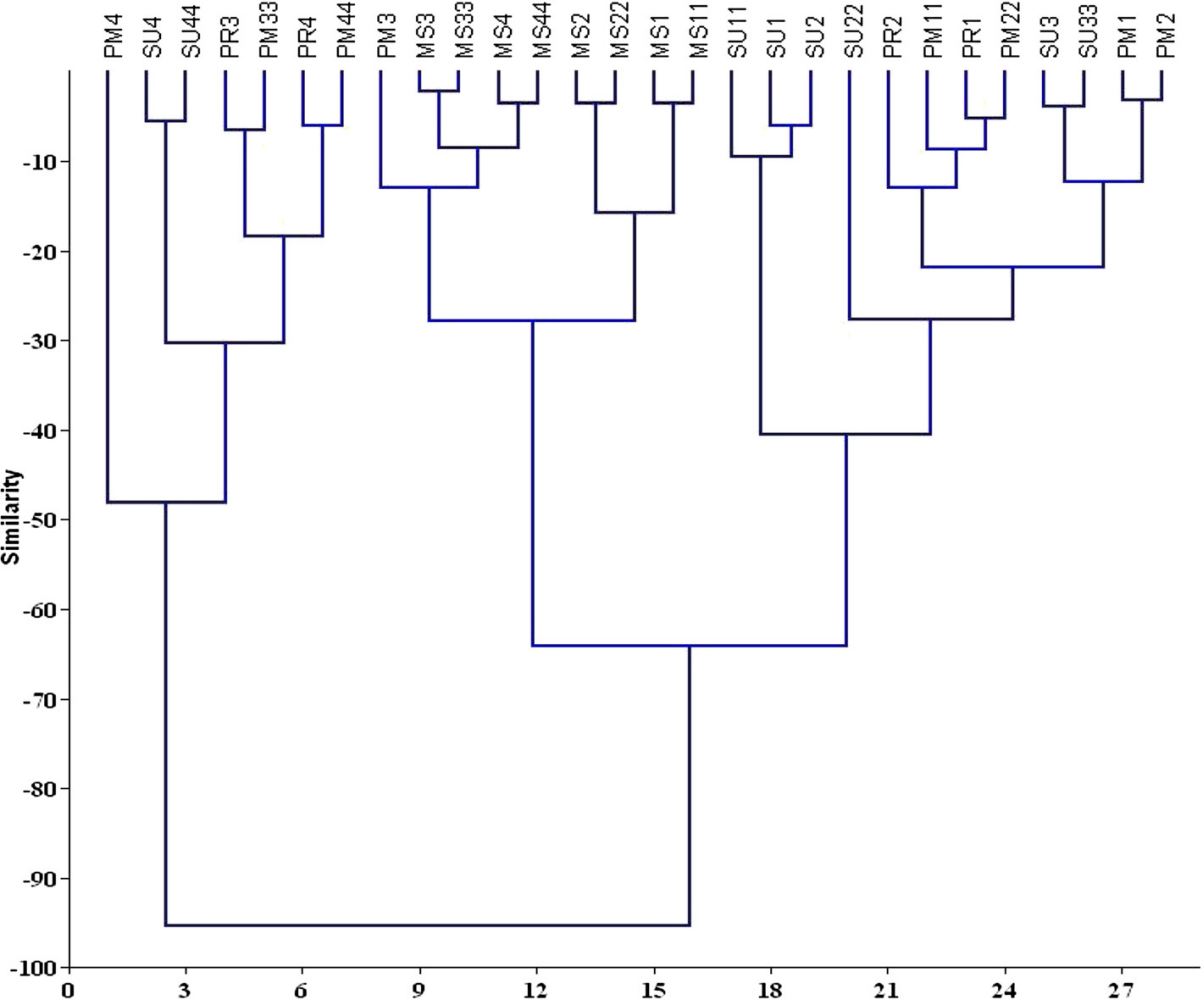
**Bray-Curtis similarity dendrogram showing grouping of stations sampled during different.** seasons for infauna MS1= Monsoon station 1 ; MS2 = Monsoon station 2; MS3 = Monsoon station 3; MS4 = Monsoon station 4; PM1= Post monsoon station 1; PM2 = Post monsoon station 2; PM3 = Post monsoon station 3; PM4 = Post monsoon station 4; SU1 = Summer station 1; SU2 = Summer station 2; SU3 = Summer station 3; SU4 = Summer station 4; PR1 = Premonsoon station 1; PR2 = Premonsoon station 2; PR3 = Premonsoon station 3; PR4 = Premonsoon station 4; MS11 = Monsoon station 11; MS22 = Monsoon station 22; MS33 = Monsoon station 33; MS44 = Monsoon station 44; PM11 = Post monsoon station 11; PM22 = Post monsoon station 22; PM33 = Post monsoon station 33; PM44 = Post monsoon station 44; SU11 = Summer station 11; SU22 = Summer station 22; SU33 = Summer station 33; SU44 = Summer station 44.

**Figure 15:**
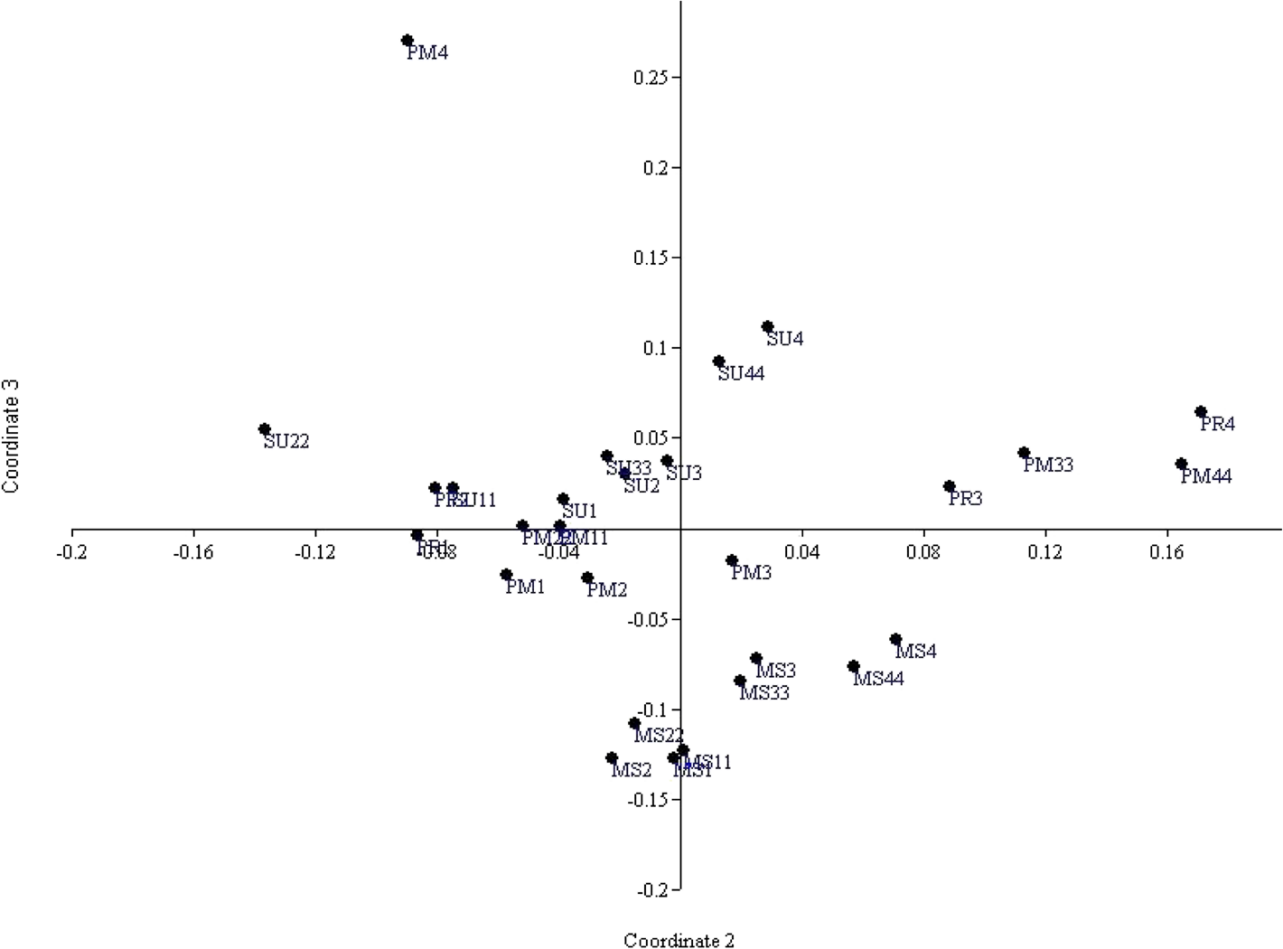
**MDS plot similarity showing grouping of stations sampled during different seasons.** MS1= Monsoon station 1 ; MS2 = Monsoon station 2; MS3 = Monsoon station 3; MS4 = Monsoon station 4; PM1= Post monsoon station 1; PM2 = Post monsoon station 2; PM3 = Post monsoon station 3; PM4 = Post monsoon station 4; SU1 = Summer station 1; SU2 = Summer station 2; SU3 = Summer station 3; SU4 = Summer station 4; PR1 = Premonsoon station 1; PR2 = Premonsoon station 2; PR3 = Premonsoon station 3; PR4 = Premonsoon station 4; MS11 = Monsoon station 11; MS22 = Monsoon station 22; MS33 = Monsoon station 33; MS44 = Monsoon station 44; PM11 = Post monsoon station 11; PM22 = Post monsoon station 22; PM33 = Post monsoon station 33; PM44 = Post monsoon station 44; SU11 = Summer station 11; SU22 = Summer station 22; SU33 = Summer station 33; SU44 = Summer station 44.

Cluster analysis revealed three distinct benthic macroinvertebrate fauna groupings, which appeared to reflect differences in sediment/habitat types within Pondicherry mangroves (Figure 14). Cluster 1 consisted of Stations 1 and 2, outside of the monsoon season, with high diversity, richness and abundance of organisms, particularly *C. cingulata; T. telescopium, Anadara rhombea* and *M. meritrix.* Cluster 1 was also characterized by high proportion of coarse sediments and high DO levels. From the resulting dendrogram, it was possible to grade the results according to stations and seasons. They consisted Cluster −1 (PM1, PM2, PM11, PM22, PR1, PR2, SU1-SU3, SU11-SU33), Cluster 2 consisted of all stations during the monsoon season, which is a period characterized by high rainfall and river flow. Cluster 3 consisted of Stations 3 and 4 outside of the monsoon season and was characterized by low species abundance and diversity, along with high organic matter (OM) and sulphide content. Cluster 3 also contained pollution indicator species *C. nucleus, M. ceylonicaus*, and *S. minuta.* An MDS plot based on the average abundance of benthic macroinvertebrate fauna and environmental parameters revealed three distinct groups at 52% similarity (Figure 15). Group 1 included Stations 1 and 2, and was characterized by the dominance of *C. cingulata, Perna virdis, Uca annulipes* and *Uca* sp. Group 2 included Stations 3 and 4 and was characterized by high OM and sulphide concentrations. Group 3 contained all values from all sites during the monsoon season.

**Figure 16:**
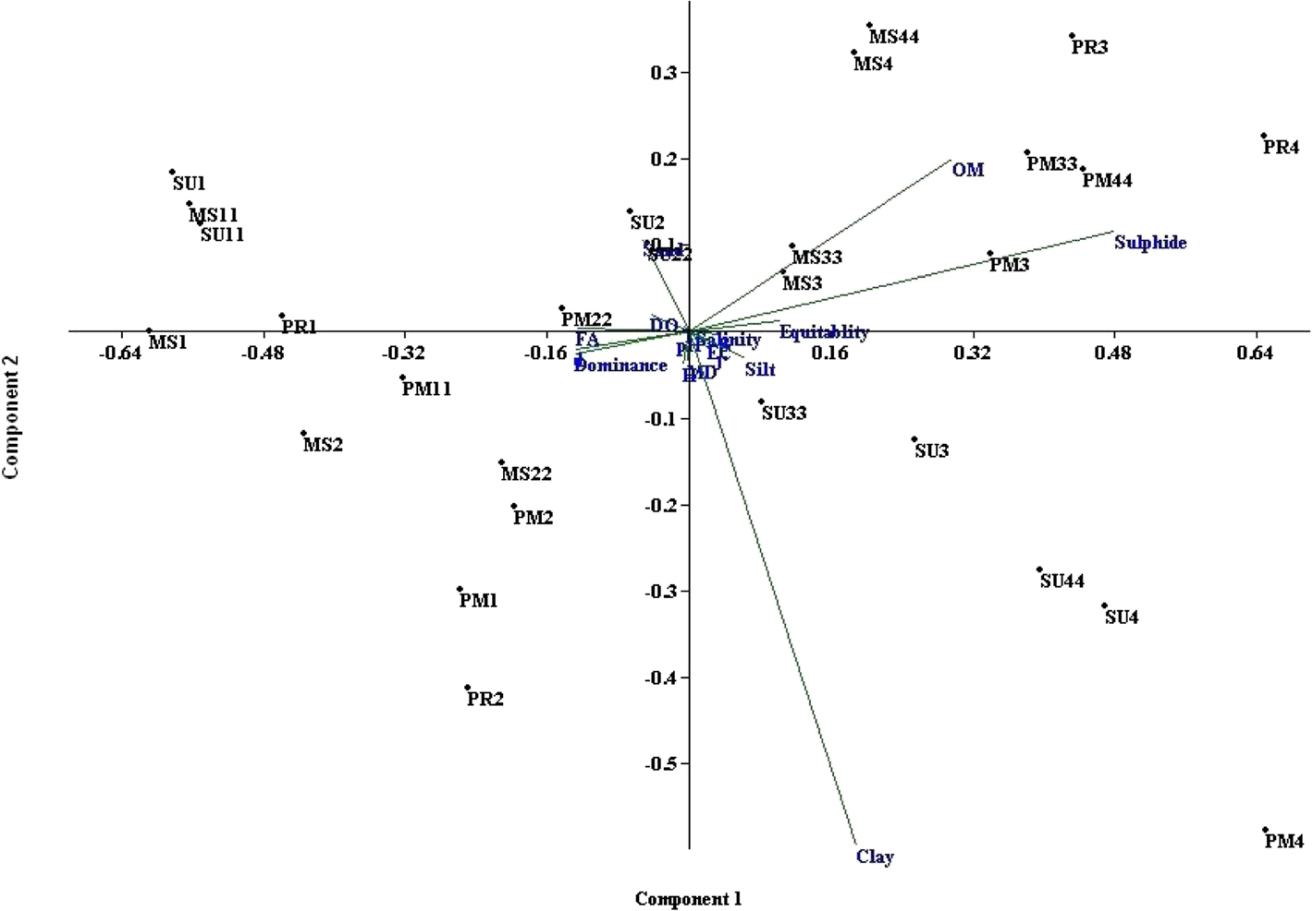
**Principal Component analysis showing grouping of stations sampled during different seasons.** MS1= Monsoon station 1 ; MS2 = Monsoon station 2; MS3 = Monsoon station 3; MS4 = Monsoon station 4; PM1= Post monsoon station 1; PM2 = Post monsoon station 2; PM3 = Post monsoon station 3; PM4 = Post monsoon station 4; SU1 = Summer station 1; SU2 = Summer station 2; SU3 = Summer station 3; SU4 = Summer station 4; PR1 = Premonsoon station 1; PR2 = Premonsoon station 2; PR3 = Premonsoon station 3; PR4 = Premonsoon station 4; MS11 = Monsoon station 11; MS22 = Monsoon station 22; MS33 = Monsoon station 33; MS44 = Monsoon station 44; PM11 = Post monsoon station 11; PM22 = Post monsoon station 22; PM33 = Post monsoon station 33; PM44 = Post monsoon station 44; SU11 = Summer station 11; SU22 = Summer station 22; SU33 = Summer station 33; SU44 = Summer station 44.

In the PCA analyses, variables associated with principle components 1 and 2 accounted for 70.3% variability among the samples (Figure 16). Stations 1 and 2 grouped on the left side of the plot (correlating with high abundances of macro benthic organisms, high salinity and DO, and low sulphide and OM concentrations). Stations 3 and 4 grouped together on the plot (correlating with low abundances of macro benthos organisms, low DO and salinity, and high sulphide and OM levels). In the multivariate analysis, monsoonal samples were ordinated separately from all other samples.

## Discussion

### Water quality parameters

Variations observed in water quality parameters can be attributed to regional patterns in climate and biological activity. Generally, surface water temperature is influenced by the intensity of solar radiation, evaporation, freshwater influx and cooling and mixing up with ebb and flow from adjoining neritic waters [45]. In the present study, summer peaks and monsoonal troughs in air and water temperatures were similar to those reported in the east and west coast of India [45]. Salinity acts as a limiting factor in the distribution of living organisms, and its variation caused by dilution and evaporation influences faunal distribution in the intertidal zone [46]. In the present study salinity at all the four stations was high in summer and low in the monsoon season indicating that variation in salinity at study sites was affected by freshwater runoff entering the creek systems, as observed in the Gulf of Kachchh [47] and Uppanar estuary [48]. Hydrogen–ion concentration varied little among the four stations and pH was alkaline throughout the study period. Higher pH observed in summer season could be attributed to the removal of CO_2_ by the photosynthetic organisms and the lower pH during monsoon season could be due to the dilution of saline water by freshwater inflow [47]. The relatively low DO values observed in the summer are attributable to the prevalence of high saline neritic waters in the mangrove channels, as well as fluctuations in temperature and salinity, which affect dissolution of oxygen [49]. The low DO levels recorded at Stations 3 and 4 may be attributable to the inflow of pollutants and high oxygen demand from elevated levels of hydrogen sulphide. Higher sulphide levels at Station 3 and 4 are in part related l to industries discharging effluents near these two stations. A peak value of H_2_S (0.92 μg g-1) in soil has been reported at Kayamkulam estuary [50]. The presence of high sulphide content in the sediments of polluted regions in this study is likely one of the limiting factors in benthic macroinvertebrate fauna abundance and distribution. By contrast sediments with a mixture of organic matter, sand and clay, but low sulphide, seemed to support higher abundances of benthic marco fauna. Distribution and ecology of benthic communities in relation to station and season.

This study provides a baseline for the distribution, abundance and diversity of benthic macroinvertebrate fauna of Pondicherry mangroves in India. The order of importance of mollusks (gastropods and bivalves), crustaceans, amphipods and polychaetes in the present study is similar to that observed by [51]. Macro benthic faunal densities observed in this study (140–1113 ind.m-2) were higher than that reported by [52] in Zuari estuary (50–1037 ind.m-2) and [53] in Andaman seas (80–998 ind.m-2). The densities were comparable to those observed in the gulf of Arid Zone mangroves of Gulf Kachchh on the west coast of India (i.e. 424–2393 ind.m-2) [8]. The high densities recorded in the post-monsoon season in Pondicherry mangroves could be due to low temperatures and turbidity coupled with stable environmental conditions. Post-monsoon season (Nov-Feb) peaks in density have also been reported for the west coast of India [51].

In the present study, 22 species belonging to 12 genera and 5 families of brachyuran crabs were recorded in Pondicherry mangroves. The distribution of crabs showed relationships to substratum characteristics, salinity, degree of tidal inundation and wave exposure. The distributional patterns are significant from a harvest perspective. *Uca annulipes, U. inversa,* and *U. triangularis* are largely caught during the monsoon season. *Scylla serrata* and *Thalamita crenata* are mostly caught during the post-monsoon season. *Portunus sanguinolentus* and *Calappa lophos* are generally caught mouths of mangrove regions.

In Pondicherry mangroves 16 bivalves and 19 gastropods were recorded, similar to the numbers previously reported for mangroves of Sunderbans [4], Saravanakumar [8]. Pollution indicator species, such as *C. nucleus, N. violacea, M. ceylonicus, and S. minuta* were observed at the two most impacted regions, i.e. Stations 3 and 4. *Modiolus metcalfei*, *C. obtusa, Cantharus tranquebaricus* are found more during the post-monsoon season at mud and sand flats, especially near the estuary mouth. *C. tranquebaricus* was reported for the first time in Pondicherry mangroves southeast coast of India.

Schrijvers et al. [54] reported the dominance of polychaetes over mollusks in Kenyan mangrove fauna due to silty clay substratum. Soft mangrove substrates favor tube dwellers over diggers and burrowing animals, such as bivalves. The high diversity of polychaete indicate favorable ecological conditions that exist in mangrove ecosystems [55]. Benthic macro fauna are important components of coastal food webs. Many fish species in estuarine ecosystems are strongly dependent taxa available that reside on the sediment surface, such as amphipods, mysids, and surface deposit feeders (SDF) [56]. Several studies have demonstrated the presence of amphipods in Pitchavaram mangroves of India [5,57]. In the present study seven amphipod species were observed. Due to limited dispersion capabilities and habitat specificity of amphipods, they may be of use in biogeography and environmental monitoring of mangrove ecosystems.

Limited quantitative information has been published on mangrove habitats in India, including the distribution of benthic macroinvertebrate fauna and their relationship to environmental factors [3-10]. The results of cluster analysis in this study show three major groupings, mainly segregated by region and season. Stations 1 and 2 clustered together in all but the monsoon season, largely based on the high density and diversity of organisms. The increased abundance of organisms, species richness and diversity observed in Stations 1 and 2 could be due to the presence of coarse sediments, high DO and salinity [58,59]. Stations 3 and 4 clustered together based on low abundance and diversity, perhaps due to the silty characteristics of the sediments, along with high organic matter content, sulphide concentrations and low DO [16]. From a seasonal perspective, all sites clustered together during the monsoon season, demonstrating the impact of climatic conditions.

### Species diversity indices

Pearson and Rosenberg [11] proposed that diversity indices provide important insights into faunal communities at different stages in succession. Snelgrove [60] proposed that the species diversity is mainly controlled by fluctuations in the environment that leads to less diversity. Species diversity tends to be low in physically controlled ecosystems [61]. In the present study, species richness of benthic macrofauna was highest during the post-monsoon season and summer, similar to observations from Cochin backwaters [62]. Low population density recorded in the monsoon season was apparently due to the effect of heavy rainfall, as previously observed by Saravanakumar et al. [8], whoireported a ‘severe decline’ of macro benthos in the shallow waters during southwest monsoon attributed to lowered salinity [51].

Wilhm and Dorris [63] propose that values less than 1.0 for diversity index (*H*) in estuarine waters indicate heavy pollution, values between 1.0 and 3.0 indicate moderate pollution, and values exceeding 3.0 indicate non-polluted water. Diversity values in the study area ranged from 1.83-2.83. Thus, these values suggest that the mangroves examined in this study are moderately polluted and the macro benthic community is under stress due to natural and/or anthropogenic factors. Maximum diversity and richness recorded in this study during the post-monsoon season might be due to stable environmental factors, such as high DO and salinity, which play a vital role in faunal distribution. Relatively high species richness, evenness and diversity were observed at Stations 1 and 2, compared to Stations 3 and 4. The low species richness recorded in this study during monsoon might be due to the freshwater runoff containing inadequately treated sewage and low salinity, which in turn affected the distribution of benthos.

### Multivariate statistics

CA, MDS and PCA have been widely used in the evaluation of spatial and temporal variations in water quality and benthic characteristics of aquatic ecosystems [35,42,64]. CA was used in this study to examine the differences between the monitoring stations during the four seasons of the year. Based on the cluster analysis, the concentration of sulphide and organic matter in cluster 3 (i.e. Stations 3 and 4) were high compared to clusters 1 (Stations 1 and 2) and 2 (all sites during the monsoon season). In defining environmental factors important in characterizing mangrove condition MDS analyses were used. Low DO, high sulphide, low salinity, high OM and high clay content appear to indicate deteriorating water quality from the standpoint of benthic macroinvertebrate fauna. Human activities have a strong influence on the aquatic environment in the southeast coast of Bay of Bengal [45]. Sources of pollution include agricultural runoff, leaching from solid waste disposal sites, domestic and industrial waste disposal. Similar approaches based on Results of multivariate analysis suggests that for mangrove benthic macroinvertebrate fauna that environmental parameters such as DO, sulphide, salinity, OM and clay have a strong influence on species composition and diversity.

## Conclusions

This study provides insights into the effects of a range of environmental parameters on macro benthic communities of Pondicherry mangroves in India. Altogether 76 species of benthic macroinvertebrate fauna, belonging to five major groups, were identified at the four sampling stations. Station 1 was dominated by sandy sediment, high salinity, high DO and relatively low sulphide levels. The region displayed the high species diversity, abundance and species richness. Station 2 was characterized by sandy sediment, low organic matter and relatively high species diversity. Stations 3 and 4 had higher sulphide concentrations, silty sediments and lower DO, with relatively low species diversity. The temporal distribution of benthic macro invertebrate fauna exhibited the highest species density during post-monsoon season. The decrease of benthos during the monsoon may be attributable to low temperatures and salinities. CA, MDS and PCA analyses were useful in helping to define spatial and temporal patterns in mangrove water quality and benthic macro invertebrate fauna characteristics in the Pondicherry mangroves. Temperature, salinity, DO, sulphide, sediment composition and organic matter content all proved to be important descriptive parameters in terms of the abundance and distribution of benthic fauna.

## Competing interests

Both authors have declared that no competing interests exist.

## Authors’ contributions

Author ABK designed the study, wrote the protocol. Author PSK carried out the laboratory work and wrote the first draft of the manuscript. Author PSK performed the statistical analysis and managed the literature searches. Author ABK supervised the overall work. Both authors read and approved the final manuscript.
